# An Information Theory-Based Approach to Assessing Spatial Patterns in Complex Systems

**DOI:** 10.3390/e21020182

**Published:** 2019-02-15

**Authors:** Tarsha Eason, Wen-Ching Chuang, Shana Sundstrom, Heriberto Cabezas

**Affiliations:** 1 National Risk Management Research Laboratory, U.S. Environmental Protection Agency, Research Triangle Park, NC 27711, USA; 2 National Research Council, U.S. Environmental Protection Agency, 26 W. Martin Luther King Drive, Cincinnati, OH 45268, USA; 3 School of Natural Resources, University of Nebraska-Lincoln, 103 Hardin Hall, 3310 Holdrege St., Lincoln, NE 68583, USA; 4 National Risk Management Research Laboratory, U.S. Environmental Protection Agency, Cincinnati, OH 45268, USA; 5 Institute for Process Systems Engineering and Sustainability, Pazmany Peter Catholic University, Szentkiralyi utca 28, H-1088 Budapest, Hungary

**Keywords:** Fisher information (FI), information theory (IT), spatial assessment, regime shifts, geospatial data, early warning indicators (EWI)

## Abstract

Given the intensity and frequency of environmental change, the linked and cross-scale nature of social-ecological systems, and the proliferation of big data, methods that can help synthesize complex system behavior over a geographical area are of great value. Fisher information evaluates order in data and has been established as a robust and effective tool for capturing changes in system dynamics, including the detection of regimes and regime shifts. The methods developed to compute Fisher information can accommodate multivariate data of various types and requires no a priori decisions about system drivers, making it a unique and powerful tool. However, the approach has primarily been used to evaluate temporal patterns. In its sole application to spatial data, Fisher information successfully detected regimes in terrestrial and aquatic systems over transects. Although the selection of adjacently positioned sampling stations provided a natural means of ordering the data, such an approach limits the types of questions that can be answered in a spatial context. Here, we expand the approach to develop a method for more fully capturing spatial dynamics. The results reflect changes in the index that correspond with geographical patterns and demonstrate the utility of the method in uncovering hidden spatial trends in complex systems.

## 1. Introduction

Today’s digital landscape presents a world full of information with more access to geotagged datasets. Accordingly, in this era, analysts are less likely to struggle with a lack of available data. Instead, they are taxed with overwhelming amounts of information and charged to make good use of the data. Large-scale datasets present a variety of challenges (e.g., storage, data integrity, security), yet offer great opportunities for pivotal discoveries. From detecting medical outbreaks to mining social media data to developing management options for impaired ecosystems, there is a great need for methods that not only provide insight on observable phenomena but can uncover latent characteristics and emergent properties in a veritable data haystack [1].

Geospatial assessment is a well-developed and growing field. Spatial analyses typically involve the visual assessment of mapped parameters, use of zonal/spatial statistics (e.g., Moran’s I), or regression and data aggregation approaches (e.g., principal components analysis) [2,3]. Visual analytics, data mining and data discovery are fields in statistics catalyzed by high speed computing, enhanced data storage capability, machine learning techniques and visualization tools [4,5]. These approaches are often highly interactive, and applications may range from simple data exploration and visualization to pattern recognition and model development. They have been used to study a variety of problems from land use change and text mining to intelligent transportation and network security (e.g., [6,7,8]). Szewrański et al. [9] demonstrate the utility of combining GIS and business intelligence (BI) to enhance visual data discovery by linking ArcGIS and Tableau. Similar to the use of ArcGIS with programming tools (e.g., R, Python) or BI platforms (e.g., Microsoft Power BI, Qlik Sense), such an approach capitalizes on the unique strengths of each tool. While this is a useful approach, researchers note that complex problems often necessitate the use of highly complicated tools and techniques which may limit a broader application of the approaches [4,10]. Still researchers are faced with the need to understand complex systems, capture patterns and trends in multiple variables, and identify system drivers. Furthermore, there is a growing emphasis on identifying patterns in underlying dynamics before a system shifts in its overall condition, which can result in costly, long-term effects. A large and thriving literature presents the development and use of statistical approaches to detect early warning signals of regime shifts and tipping points in time series data, but there is a relative lack of such studies on spatial regimes.

Classic early warning indicators (EWI) are based on the concept of critical slowing down (CSD), the phenomenon whereby a system’s rate of return to equilibrium slows down in the proximity of a bifurcation point [11,12]. These CSD-derived indicators assess univariate data for changes in variance, autocorrelation, conditional heteroskedasticity, density ratio and spectral reddening, among others [13,14]. Their appeal lies in their generality and ability to be widely applied without requiring equations, models, or even a mechanistic understanding of the key system processes. However, when applied to real data, inconsistencies in the ability of CSD-derived indicators to detect regime shifts have been problematic [15,16,17,18,19]. Their general applicability was also reduced when researchers found that not all bifurcations are preceded by CSD [20], giving a false negative, and that it is possible to detect critical slowing down in systems that exhibit nonlinearity but do not have a bifurcation point, giving a false positive [21]. Spatial correlates of CSD-derived indicators (e.g., spatial variance, near-neighbor autocorrelation, spatial skewness and spatial spectral density) have been developed and offer many of the same benefits and fewer concerns than their temporal correlates [11,22,23]. Their utility is being confirmed in empirical studies [24,25,26,27], but their trends may not be consistent in self-organized, patterned spatial systems because environmental changes other than an impeding regime shift may be driving trends in the indicator [28]. Spatially heterogeneous stressors also appear to confound the detection of a CSD-signal [29,30]. 

Alternative spatial EWIs that aim to avoid the issues associated with CSD-derived indicators have largely been based on vegetative patch size distributions, with the expectation that they fit a power law function unless an environmental stressor changes the patch size distribution by truncating it [31,32]; thus, a changing power law fit acts as an EWI. There has been controversy over the biological reasonableness of this approach [33]. Regardless of the merits of the debate, the method was developed for terrestrial drylands, so it may not be appropriate for other types of spatial systems, particularly if they are not heterogeneously distributed across space. Rather than track the patch size distribution, several methods focus on other patch size properties such as time fluctuations in the largest cluster size, variance in the size of the largest patch in proportion to the area of the system, variance in the proportion of the largest patch to the total area occupied by the same species, and the probability that a cluster will grow or shrink as a function of its size [34,35,36,37]. However, as with the spatial correlates of critical slowing down, most authors are evaluating these variables over time, requiring temporal data to document the changes to spatial metrics [38]. Few methods can detect a critical transition with only 2–3 temporal snapshots, as Weissmann et al. [36,37] attempted with their model of probability of cluster growth.

Other spatial EWIs are being developed, such as the recovery length method of Rindi et al. [39], and network-based indicators such as degree, assortivity, and clustering [40]. The recovery length refers to the spatial distance from a perturbation at which a population recovers and may be less data intensive than classic indicators [41]. As a system moves closer to a critical transition, the recovery length increases. However, this metric is only appropriate for systems that have a sharp boundary between habitats, such as algal canopies, mussel beds, shallow lakes, salt marshes, and forest-savannah [39], and is not suitable for highly spatially heterogeneous systems. Network based indicators such as those in Yin et al. [40] may be more general in their adaptability to a system type but require long term data with high frequency measurements. A conclusion of most EWI studies is that multiple methods will always be required, to account for key differences between the ecosystem types and inconsistencies of the signal detection within a given indicator. Coupled with these issues is the challenge of capturing a regime shift using univariate data (monitoring one variable). Unless the system is exquisitely well understood, there is the risk that the variable chosen to represent the system’s response to a perturbation is insufficient or inaccurate; this is a core issue for traditional indicators [42]. Using traditional EWIs for multivariate systems requires tracking trends in the indicator separately for each individual variable (i.e., examining 50 bird species requires the computation and tracking of 50 variance patterns). However, there has been limited success with such an approach. Although Litzow et al. [43] found that monitoring an increasing variance in pooled fisheries catch data greatly increased the detection of a collapse, other researchers noted inconsistent trends in univariate EWIs (e.g., variance, autocorrelation) as a system approaches a critical transition [13,19,44]. 

Multivariate methods thus become highly desirable, as they are more likely to capture the realistic complexity inherent in human and natural systems [12,16,45]. The variance index was developed by Brock and Carpenter [46], and it detects the dominant variance component in a multivariate system. It is computed using the largest eigenvalue of the covariance matrix and should spike prior to a transition; however, the results from this index are sometimes unclear [16,17].

Information theory (IT) may offer a useful alternative to the methods mentioned above. IT-based approaches have been useful for understanding ecosystem function, structure and complexity [47,48,49,50]. In a spatial context, entropy has been applied to geography, geoinformatics (e.g., for city zoning, visualization and modelling), landscape diversity and cognitive development [51,52,53,54,55,56]. Fisher information has been demonstrated as an effective tool for capturing trends in complex systems. It can be employed to assess univariate and multivariate systems using a variety of data types (e.g., economic, social, environmental). There is no strict data requirement, minimal assumptions are necessary, and it is agnostic with regards to the degree of heterogeneity it can handle [57,58,59]. 

Fisher information was developed as a measure of disorder in data [60] and provides a means of quantifying organizational dynamics in complex systems [61]. It has been adapted into an index that reflects the dynamic order within a system by collapsing patterns in the underlying system variables into a measure that can be tracked to assess systemic change [58]. This form of Fisher information has been used to assess sustainability, political instability and resilience, and it has been proposed as an EWI in a variety of human and natural systems at multiple spatial scales (e.g., [17,57,59,62,63,64,65,66]). However, it has primarily been employed to evaluate temporal dynamics with time as a natural ordering parameter. In the first foray into geospatial assessments, Sundstrom et al. [67] used Fisher information to assess spatial regimes in avian and zooplankton communities. Abundance data was gathered from historical records for over 200 species collected from routes along transects through multiple terrestrial ecoregions and aquatic domains. The Fisher information detected spatial regimes in both systems and delivered additional details about changes in the communities not provided by other multivariate approaches. Selecting adjacent routes along each transect afforded the ability to use linear proximity (i.e., the next station) to order the data; however, such an approach limits the types of questions that can be explored or the assessments that can be performed. 

Here, our goal is to adapt the computation of Fisher information to develop a general method for handling geospatial data in a way that does not require conceptualizing the study area as a series of transects. The approach intends to offer an assessment of patterns across a landscape by capturing the trends in the variables that characterize the condition at each sampling location. Using simulated and real data, we test the utility of the method and identify mechanisms for detecting signals of geospatial change. This effort is an extension of the spatial regimes work [67] and involves examining methods at the nexus of information theory, systems thinking and geographical information systems.

## 2. Materials and Methods 

### 2.1. Fisher Information

Fisher information was developed as a statistical measure of the amount of information inherent in data useful for estimating a parameter [60]. Accordingly, it relates to the order and, therefore, the patterns in data [17]. The form of Fisher information used in this work is based on the probability of observing states (*s*) of a system, *p*(*s*) [61,68]. From Equation (1), note that the Fisher information (*I*) is proportional to the slope of the probability of observing a system state *p*(*s*) with respect to the state (*dp*(*s*)/*ds*); hence, the higher the probability of observing a state (i.e., more consistent patterns), the higher the Fisher information:

(1) $$I=\frac{ds}{p\left(s\right)}{\left[\frac{dp\left(s\right)}{ds}\right]}^{2}$$

System states reflect the system condition using a set of measurable variables (*x_i_*). When assessing temporal trends, the trajectory of a system is defined by a series of points over time, e.g., $pt_{i}\left(t_{j}\right):\left[x_{1}\left(t_{j}\right),\;x_{2}\left(t_{j}\right),\;x_{3}\left(t_{j}\right),\ldots ,x_{n}\left(t_{j}\right)\right].$ Systems may experience a nominal variation within a particular state or dramatically change due to internal dynamics (e.g., variation in linked mechanisms or in response to external perturbations). Given measurement uncertainty and the fact that systems randomly fluctuate, the points within a finite range may be viewed as observations of the same state; hence, the likelihood of a specific state relates to the number of points that fit within a specified range (or tolerance) [58]. Karunanithi et al. [58] adapted Equation (1) to handle empirical data using this grouping strategy or “binning” approach, and Fisher information (henceforth, denoted as FI) is numerically estimated as:

(2) $$\mathrm{FI}=4\overset{n}{\underset{s=1}{\sum }}{\left[q_{s}-q_{s+1}\right]}^{2}$$

where *q*(*s*) ≡ √*p*(*s*).

Interpreting FI is predicated on the fact that distinct processes and patterns control different system regimes. Since the deviations in FI indicate changes in the system condition, tracking FI provides a means of capturing this behavior. Increasing FI signifies a rising dynamic order and suggests possible movement to more consistent (stable) patterns. Conversely, decreases in FI denote instability, resilience loss and may warn of an impending regime shift [16,58,66]. When comparing the stability of different systems, regions or periods of interest, the mean (μFI), standard deviation (σFI) and coefficient of variation of FI (cvFI) may be used to help distinguish stable regimes from critical transitions (or regime shifts). Stable regimes are defined by relatively high FI with little to no variation (↑μFI and ↓σFI) [62,69]. The coefficient of variation $\left(\frac{\sigma }{\mu }\right)$ is a measure of the dispersion around the mean and is typically low for more stable systems (↓cvFI; [67]). Although transitions may be defined as declines in FI between two stable regimes [58], we identify them as periods characterized by a relatively high standard deviation and coefficient of variation in FI (↑σFI, ↑cvFI; [67]). The details on the derivation, calculation and interpretation of FI may be found in [58,61,67,70,71].

For temporal studies, the basic steps for computing FI include: (1) gathering measurable variables for the study period; (2) dividing the time series data into moving windows that advance forward one-time step for each iteration. The size of the window is based upon the amount of data; however, it is suggested that each window contain at least 8 points [70]; (3) determining the measurement uncertainty for each variable (size of states), which becomes the boundary (tolerance) around each system state. The size of states (sost) may be estimated by using the amount of variation in a stable portion of the study dataset or within a similar system as a proxy [70]; (4) in each window, binning points in states of the system using sost; (5) counting the number of points grouped in each state and dividing this value by the total number of points in the window to produce *p*(*s*); (6) computing *q*(*s*) = √*p*(*s*) and calculating FI using Equation (2). This process is repeated to provide a FI result for each window, thereby producing index values over time. Using the binning approach, FI ranges from 0 to 8 [58]. The algorithm has been coded in Matlab and Python [1,70]. 

### 2.2. Assessing Geospatial Patterns with FI

Adapting FI to assess spatial dynamics involves first understanding that the core of the approach involves tracking system states. The system condition may change both temporally and spatially, where the condition at a location (*l*) is defined by Sundstrom et al. [67] as $C\left(l_{J}\right):\left[x_{1}\left(l_{j}\right),\;x_{2}\left(l_{j}\right),\;x_{3}\left(l_{j}\right),\ldots ,x_{n}\left(l_{j}\right)\right]$. Since the goal is to evaluate patterns over a geospatial area defined by latitude, longitude and possibly elevation (or depth for aquatic systems), the challenge then becomes: What ordering principle should be used for this type of data? How do we capture patterns over an entire area? 

The initial dilemma was determining the optimal way to traverse the area ensuring that all sampling stations are included in the assessment and a FI value could be assigned to specific locations over the area. We wanted to examine the data based on the proximity of the survey sites; however, separating the area into a series of transects would not afford the ability to include adjacent stations that are not on a fixed path. Furthermore, processing the data in this manner is complicated by determining where the transects begin and end. Clustering approaches provide an interesting option but would, in effect, partition the area into discrete groups, thereby limiting the assessments to “regions” (one FI value per cluster) rather than providing unique FI values for each location over the entire geospatial area. Moving window techniques or kriging (a method of interpolation to fill data gaps or rasterize one-dimensional data) require a specific data structure (i.e., evenly distributed observations). With the aim of developing a method that uses raw data, accounts for the sampling location and is robust to the resolution, quality and type of data, we opted to use a distance measure to set up moving windows for the data. 

### 2.3. Distance as an Ordering Parameter

We considered distance (*d*) metrics computed from the Pythagorean theorem and the Haversine formula. The three-dimensional Pythagorean formula (also known as the Euclidean metric) measures the orthogonal distance between two points in linear space:

(3) $$d=\sqrt{{\left(x_{2}-x_{1}\right)}^{2}+{\left(y_{2}-y_{1}\right)}^{2}+{\left(z_{2}-z_{1}\right)}^{2}}$$

The Haversine (“great circle”) formula uses spherical coordinates to account for the curvature of the Earth’s surface (principally important when covering large areas) and is particularly useful when using latitudes and longitudes (Equation (4)) [72]:

(4) $$d=2r\;\arcsin\left(\sqrt{\sin^{2}\left(\frac{\varphi_{2}-\varphi_{1}}{2}\right)+\cos(\varphi_{1})\cos\;(\varphi_{2})\sin^{2}\left(\frac{\lambda_{2}-\lambda_{1}}{2}\right)}\right)$$

Incidentally, a map or equirectangular projection of the Pythagorean formula can be used to capture the curvature, as well:

(5) $$d=r\;\left(\sqrt{{\left((\lambda_{2}-\lambda_{1})\cos\left(\frac{\varphi_{2}+\varphi_{1}}{2}\right)\right)}^{2}+{\left(\varphi_{2}-\varphi_{1}\right)}^{2}}\right)$$

In Equations (4) and (5), λ and φ are the longitude and latitude, respectively, and the mean radius (r) is approximated at 6371 km [72]. 

To examine these methods, each approach was used to compute the distance from a reference location to the location where the data was collected (i.e., survey location). We define the reference as the point closest to the origin (or the minimum latitude and longitude). We created a short algorithm to compute the Euclidean distance and used an existing function (lldistkm.m) from the Matlab file exchange to calculate both EpPythagorean and Haversine distances [73]. Compared to Haversine calculations, it is believed that the distance estimates from the Euclidean metric are computationally “light” (simpler formula); however, the computational speed was not an issue for either method as the Matlab code produced results within seconds. Since the Haversine distance is largely viewed as a very robust, “well-conditioned” approach [72], we opted to use it to order the data. Note: the Pearson correlation coefficients between the Haversine, Euclidean and the EpPythagorean formula were high and statistically significant (rho ≥ 0.99, *p*-value ≤ 0.05) for both the model and real data.

Below are the basic steps for using FI to assess geospatial data:

1. Gather data for the study area. Data should include the route (survey station) number, route location (latitude and longitude) and values for measured variables.
2. Use the latitude and longitude for each station to compute the distance from a reference location. Here, the reference location is defined as the minimum latitude and longitude from the data. The Haversine distance from the reference location is computed for all routes.
3. Order the data into a sequence of points by the Haversine distance from the reference location (from close to far).
4. Divide the data into windows which capture small geographical “sections” of the area based on the proximity to the reference station. Essentially, the first window will contain the data from the stations that are closest to the reference site. The following window will advance forward to the next closest station, and so on. As noted in Section 2.1., each window will contain at least 8 stations.
5. Estimate the measurement uncertainty for each variable (size of states) using the amount of variation in a stable portion of the study dataset or within a similar system as a proxy [70].
6. In each window, bin points into states of the system using the sost.
7. Count the number of points grouped into each state and divide this value by the total number of points in the window to produce *p*(*s*).
8. Compute *q*(*s*) = √*p*(*s*) and calculate FI using Equation (2).
9. Repeat steps 6–8 for each window.

As in temporal studies, this process results in a FI value for each window which is plotted at corresponding route locations (latitude and longitude) over the geospatial area. For this study, the data was managed in Excel, and short Matlab algorithms were developed or employed to compute the distance metrics (Equations (3)–(5)). The existing FI code [70] was used to compute FI from the data “ordered” in step 3. The visualizations of the data and results were done in Matlab (R2018b) and ArcGIS Pro.

### 2.4. Case Studies

The spatial patterns for system variables may fluctuate in a variety of ways. They may remain relatively the same, increase (or decrease), deviate dramatically from location to location (or region to region) or exhibit some behavior between these extremes. As a rudimentary test of the ability of FI to discriminate between these basic patterns, we created four spatial surfaces that were generated by simulating data to mimic variables with geospatial patterns that are homogenous (HoG), heterogeneous (HeT), symmetrically differentiated (HnH: half homogeneous and half heterogeneous) and patchy (Patch: heterogeneous patch surrounded by a homogenous surface). The data for these surfaces were generated using the ‘rand’ function in Matlab (R2018b). We also used a combination of the simulated variables to test the method for assessing spatial patterns in multivariate systems. Finally, we employed FI to examine spatial patterns in avian community structure. The breeding bird survey data on the total species richness and total population (or number of individuals) detected at each route across the state of Louisiana were gathered from the USGS North American Breeding Bird Survey (BBS) for the years 1990 and 2014 [74]. To provide a sense of how FI performs in normal and extreme cases for discrete data, we initially tested the method on both the raw (actual) data and data simulated to mimic homogeneous and heterogeneous patterns across the state. We then compared the FI results for the raw BBS data for 1990 and 2014, and evaluated the FI values against an ecoregion map of Louisiana and a USGS land cover map [75]. The ecoregion map provides a general expectation for the community structure in that avian bird communities within an ecoregion should be more similar than bird communities from different ecoregions. The ecoregion map, however, is based on the *potential* vegetation as a function of underlying geological and climatic variables, so it does not always represent the on-the-ground reality. Therefore, we also visually assessed the changes in FI against a 2001 land use map which more accurately reflects the actual habitat types across the state. These comparisons are only meant to highlight the possible utility of a spatial assessment using FI. The BBS case study presents the basic ability of Fisher information to detect broad changes in a community structure across large spatial scales where the community structure is largely expected to be spatially autocorrelated (the bird community structure in nearby sampling locations should be more similar than that in distant sampling locations), as well as broad shifts in the community structure as a result of differences between the underlying habitats in which the routes are found.

## 3. Results

### 3.1. Case Study: Simulating Geospatial Dynamics

**Figure 1:**
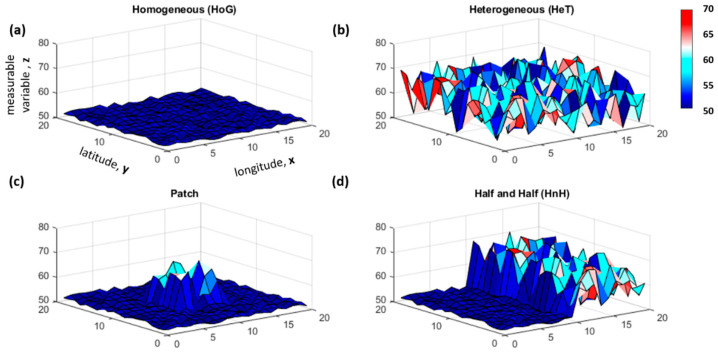
The simulated surfaces for variables representing four case study patterns: (**a**) homogeneous (HoG), (**b**) heterogeneous (HeT), (**c**) patch, and (**d**) half and half (HnH) data. The variable values range from 50 (blue) to 80 (red).

Table 1 summarizes the patterns and parameters (i.e., mean μ and standard deviation σ) used with the ‘rand’ function in Matlab to generate the data and the expected FI results for the simulated case studies. A plot of the surfaces shows that the primary axes (x, y) use cartesian coordinates from 1 to 20, and z reflects the simulated data values (Figure 1).

**Figure 2:**
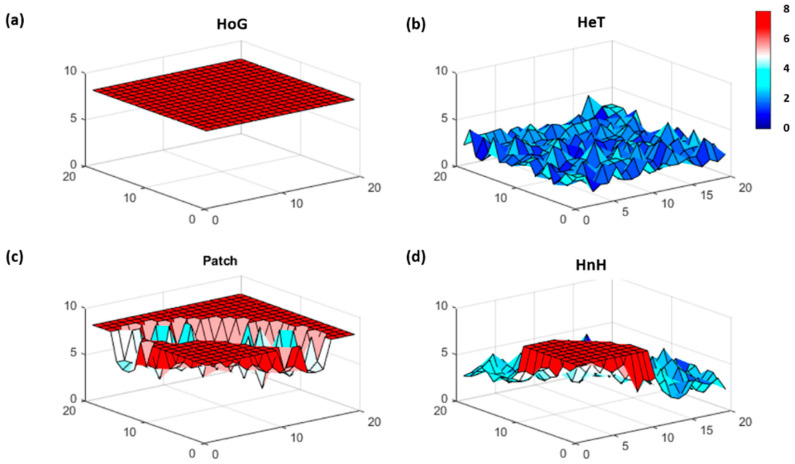
The Fisher information (FI) result for each simulated spatial pattern: (**a**) homogeneous (HoG), (**b**) heterogeneous (HeT), (**c**) patch, and (**d**) half and half (HnH) data. FI ranges from low (blue) to high (red), where the FI value at each location represents the change in dynamic order (i.e., patterns) from one location to the next. High steady FI indicates stable patterns and low FI suggests more variable patterns from location to location.

The point closest to the origin (1,1) was used as the reference point and Haversine distances were computed from this location. The survey route coordinates and variable values were ordered by the distance from the reference into a 400 X 1 array. The windows were established based on the proximity to the reference location, so that window 1 contained “stations” located at (1,1), (1,2), (2,1), (2,2), (1,3), (3,1), (2,3), (3,2), (3,3) and (1,4). Since the homogeneous patterns simulate relatively low random variation, the range of the values generated for the homogeneous case was used as an estimate of the measurement uncertainty (sost = [2]) for these initial case studies. FI values computed using a window size of 10 (hwin = 10) produced spatial patterns in line with the expected results. The homogeneous patterns reflect a high steady FI (μFI = 8, σFI = 0, cvFI = 0), and the FI for the heterogeneous case is low and noisy (μFI = 2.30, σFI = 0.69, cvFI = 0.30) (Figure 2a,b). The results for the patch (μFI = 6.91, σFI = 1.56, cvFI = 0.23), and the half and half (μFI = 4.19, σFI = 2.10, cvFI = 0.50), demonstrate how FI captures shifting spatial patterns and corresponds with changing parameter dynamics (Figure 2c,d). Furthermore, because FI is computed in overlapping windows, the trends in the index begin to change prior to “reaching” the outstanding feature (e.g., patch).

**Figure 3:**
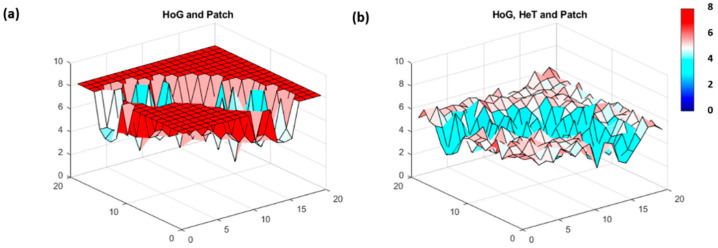
FI result for multivariate data which combine: (**a**) HoG and Patch, and (**b**) HoG, HeT and Patch. FI values range from low (blue) to high (red), where the value at each location represents the change in dynamic order (i.e., patterns) from one location to the next. High steady FI indicates stable patterns and low FI suggests more variable patterns from location to location.

The true power of FI is highlighted when the method is used to assess multivariate data. We used a combination of the simulated cases to mimic a multivariate system comprised of two (HoG and Patch) and three (HoG, HeT and Patch) variables. Note that the characteristics of the underlying variables remained intact and showed through even when in combination with other distinct patterns (Figure 3).

### 3.2. Case Study: Breeding Bird Survey Data

**Figure 4:**
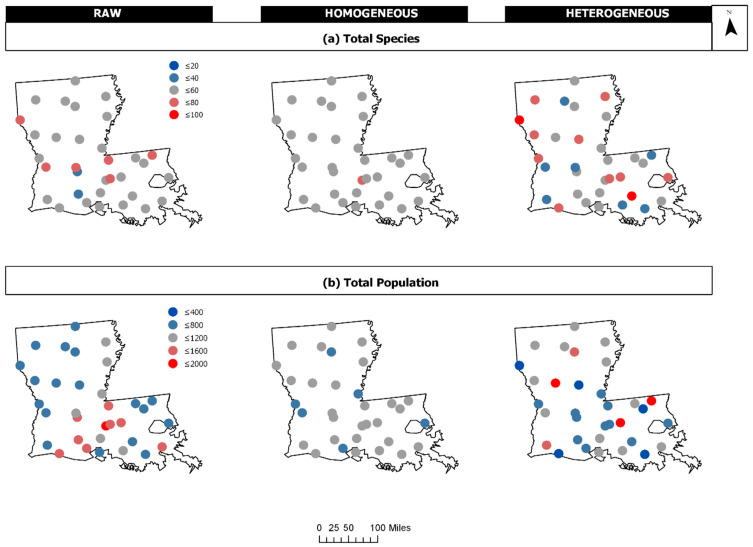
The raw Louisiana breeding bird survey (BBS) data for 1990 contrasted to simulated data representing homogenous and heterogeneous communities: (**a**) Total Species and (**b**) Total Population. The values for total species and total population range from low (blue) to high (red).

**Figure 5:**
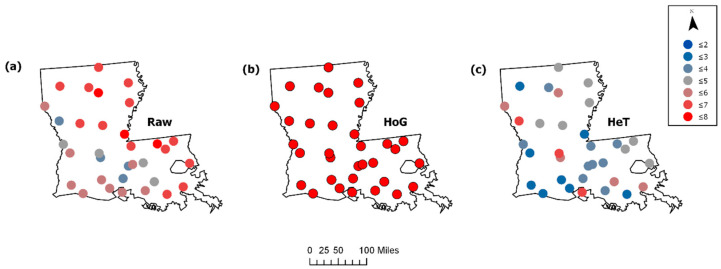
The FI results for a multivariate assessment of the bird community structure. FI was analyzed using both the total species and total population data for (**a**) raw 1990 Louisiana BBS data and simulated (**b**) homogeneous (HoG) and heterogeneous (HeT) patterns. The FI values range from low (blue) to high (red). High steady FI indicates stable patterns, while low FI suggests more variable patterns from location to location.

A comparison of the raw 1990 Breeding Bird Survey data and simulated data representing homogeneous (HoG) and heterogenous (HeT) patterns demonstrates the performance of FI on discrete data. Figure 4 provides a plot of the raw and simulated total species (TS) and total population (TP) data for each survey route. Table 2 displays the data sorted by Haversine distance (in miles) from the minimum latitude and longitude (ref = [29.55, −93.97]) to the route locations. FI was computed using a window size of 8 (hwin = 8), and the size of states was estimated based on the range of the homogeneous data (sost = [15.14 452.86]). FI values were plotted at the route locations (latitude, longitude) corresponding to each window. The results from the raw BBS data (μFI = 5.72, σFI = 1.20, cvFI = 0.23) indicate an increasing FI (and stability) at the survey routes from southwest to east across the landscape, with a clear reduction in FI from central west to southeast separating the state, as well as a high FI near the eastern border (Figure 5a). As expected, FI for the homogeneous data (μFI = 8, σFI = 0, cvFI = 0) is reflected by high steady FI, and the exact opposite is true for the heterogeneous data (μFI = 4.09, σFI = 1.14, cvFI = 0.28), where FI is relatively low and highly variable for much of the area (Figure 5b,c).

**Figure 6:**
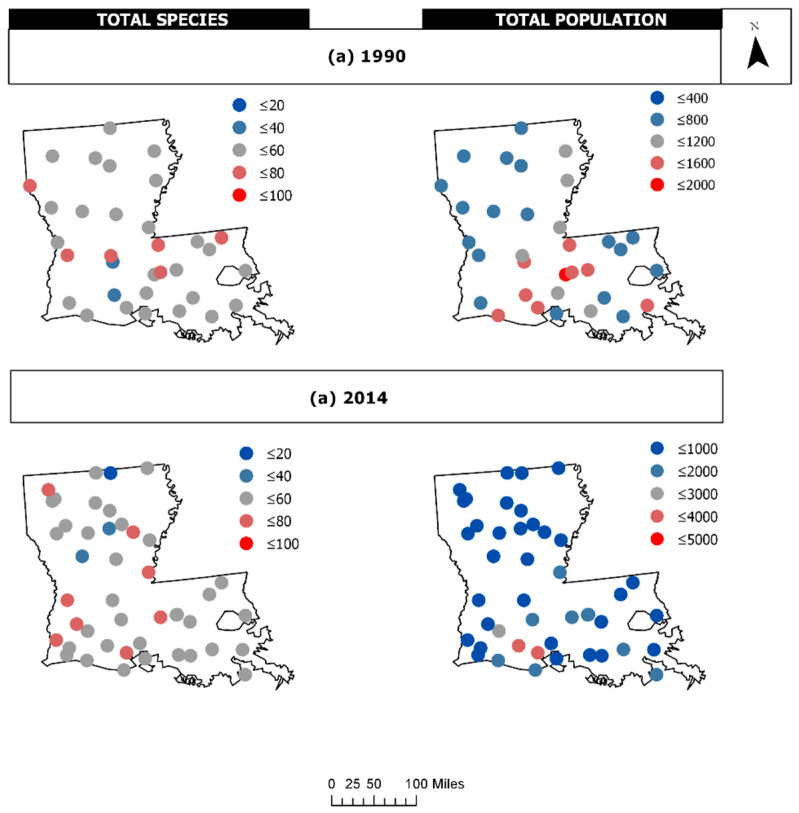
The Breeding Bird Survey (BBS) community data for (**a**) 1990 and (**b**) 2014. Total species is the number of unique species detected at each survey route location, and total population is the number of individual breeding birds detected at each route.

**Figure 7:**
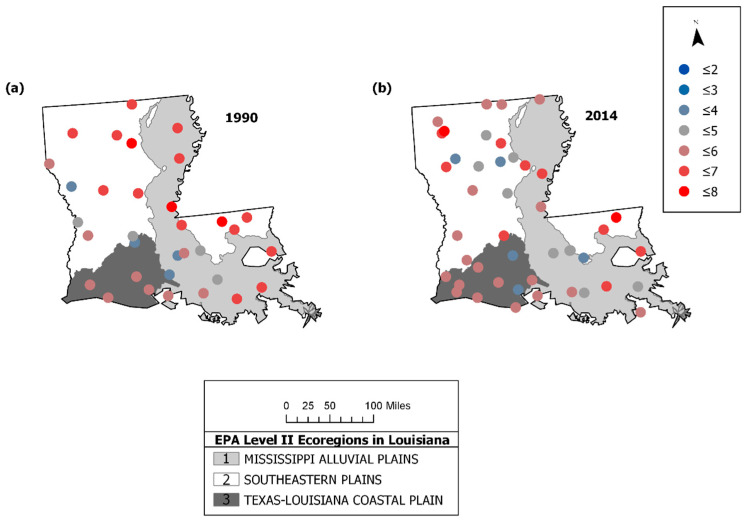
FI for the BBS community data by ecoregion [76] for (**a**) 1990 and (**b**) 2014. FI was calculated using multivariate data, consisting of total species and total population at each route location. High steady FI indicates stable patterns and low FI suggests more variable patterns from location to location.

**Figure 8:**
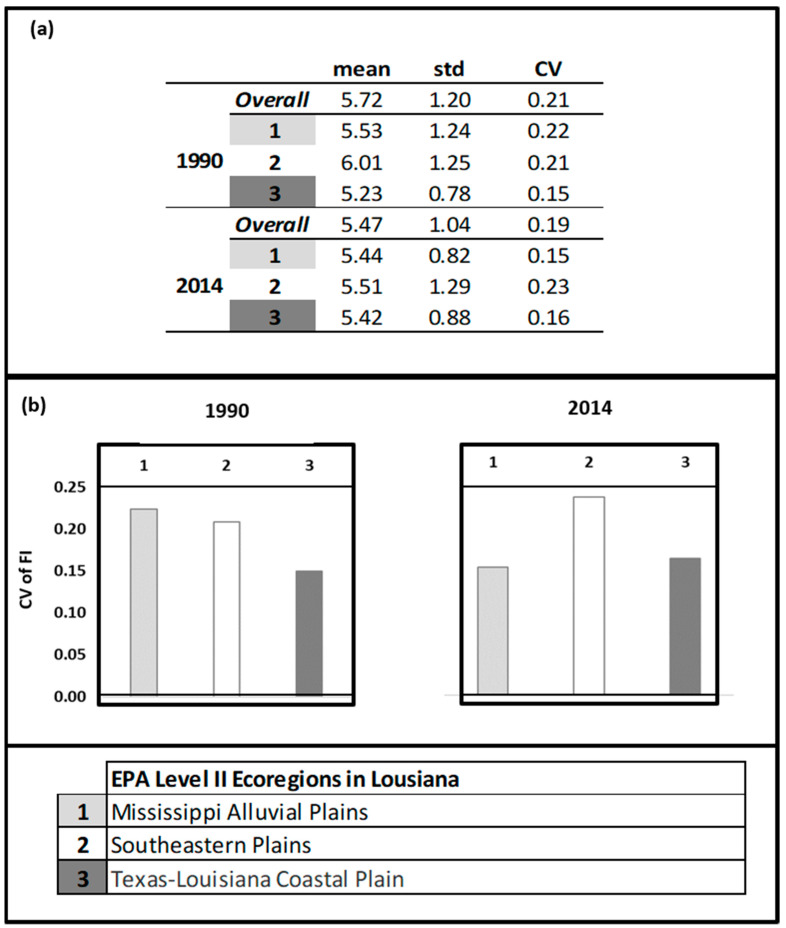
A comparative assessment of FI for 1990 and 2014 BBS data by ecoregion [76] showing (**a**) summary statistics (mean and standard deviation) for FI and (**b**) a plot of the coefficient of variation (CV) for FI. Stable regions have a relatively high mean FI, low standard deviation in FI and low coefficient of variation in FI.

**Figure 9:**
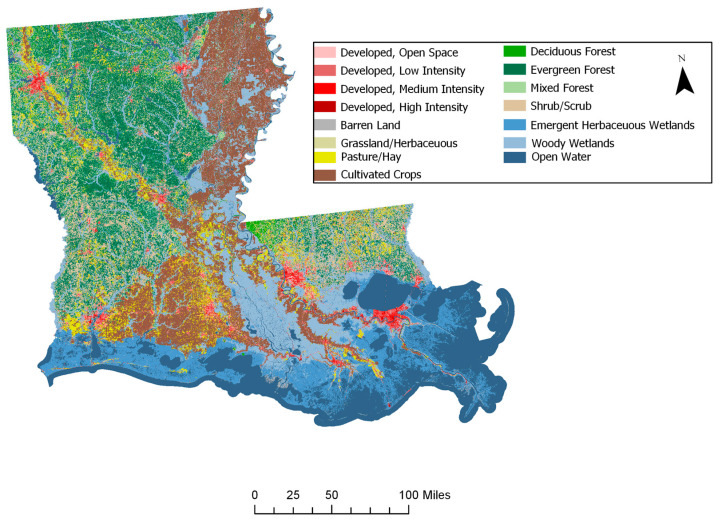
2001 Landcover data for Louisiana [75].

To compare the 1990 results to more recent trends in the avian community structure, we also evaluated 2014 BBS data. Figure 6 provides a plot of the raw data for 1990 and 2014, showing distinctive patterns across the state. Note that although the average number of species and total population (number of individuals) were quite similar between the years (1990: (53.09, 906.18); 2014: (53.23, 941.57), respectively), the maximum number of individuals sampled at the routes exhibited over a 2-fold increase from 1990 to 2014 (from 1620 to 3987 individuals); hence, there was much more variability in the 2014 avian population (σ_1990_: 354.28, σ_2014_: 757.89). The survey routes fell into three ecoregions, the Mississippi Alluvial Plains, the Southeastern Plains, and the Texas-Louisiana Coastal Plain. The patterns of stability appear to have shifted over time (Figure 7). The avian community structure in the Mississippi Alluvial Plains increased in stability from 1990 to 2014 (cvFI: 0.22 to 0.15). The Texas-Louisiana Coastal Plain had the most stable FI in 1990 (cvFI = 0.15) and maintained a similar level of stability in 2014 (cvFI = 0.16). The Southeastern Coastal Plains remained highly variable for both years (Figure 8a,b). An examination of the land use map (Figure 9) provides a visual assessment of the degree of habitat heterogeneity within each ecoregion, and roughly confirms these findings. For example, the Southeastern Plains consists of a heterogenous intermixture of pasture/hay and medium intensity human developments in woody wetland/forest, whereas the Texas-Louisiana Coastal Plan is more homogenous and largely dominated by cultivated crops and pasture/hay.

## 4. Discussion and Concluding Remarks

With the rise in availability of large-scale geospatial datasets coupled with the complexity of challenges in a more connected global society, there is a need for methods that afford the ability to examine patterns and trends in multiple variables without requiring the use of modelling, restrictive methods or stringent data requirements. Fisher information has been used to study patterns in a variety of human and natural systems. Researchers have effectively demonstrated the utility of the method and compared it to contemporary approaches, noting that the approach often delivers unique information regarding the patterns of change in complex system dynamics not present in other methods [17,67]. While it has been used to explore temporal change in social and ecological systems of various scopes and scales, its limited application to spatial data showed promise [67]. To examine such data, it was necessary to adapt the method to capture the dynamic order over a geospatial area. The previous version of Fisher information was constrained because the approach involved ordering data along one dimension. In other words, the data was either ordered by time (e.g., [17]), or by using geographically sequential sampling locations that fell along a “straight-line” transect [67].

To develop a means of assessing the dynamic order in a spatial context, we considered a variety of methods, including a cluster analysis and complex moving window techniques. However, upon revisiting the theory and framing the quandary in its most basic terms (changing condition from location to location), we found a simple solution: order the sampling locations by distance. Euclidean and Pythagorean metrics are well-known approaches. However, because the methods measure orthogonal distances, they produce “errors”, particularly when approaching meridians [72]. The Haversine formula accounts for the curvature of the Earth’s surface and is generally seen as the most efficient method for assessing distance based on latitude and longitude; accordingly, it was used for the analyses. As a side note, we found that the equirectangular projection of the Pythagorean distance provided an approximation that closely resembled the Haversine results, and the distance computed from all three methods was highly correlated.

Distance as an ordering parameter was quite useful for adapting FI for spatial assessments. The approach afforded the ability to use moving windows (which capture small geographical sections of data) to traverse the geospatial area by organizing the data based on distance from a reference location. The case study results for the simulated data reflected changes that corresponded with geographical dynamics and matched the expected results based on an understanding of patterns from previous FI work (e.g., [77]). FI from the breeding bird case study highlighted multiple ways in which the method could be useful for spatial assessments: to monitor change over a geographical area, within a spatial region, or even to compare homogeneity/heterogeneity among regions. In addition, the method could be used in longitudinal studies to determine how patterns changed over time (e.g., pre- and post-hurricane Katrina). Due to changes in the sampling techniques, resources, catastrophic events, topographical changes, etc., it is not uncommon for sampling sites to vary over time. Typically, alternate locations are chosen which capture important variables at sites that are accessible to surveyors and adequately cover particular regions or features of interest. Spatial Fisher information computations are based on the data collected at sampling sites, and while the approach is not limited by static sampling locations, as with any method, it is important to ensure that the survey sites available during the periods of interest capture the same area. As demonstrated by the comparative assessment of breeding bird survey patterns in 1990 and 2014, note that while the sampling sites were not exactly the same in both years, the locations still covered the same area. In addition, the number of survey sites actually increased from 33 in 1990 to 44 in 2014. Still, we were able to comparatively assess how a breeding bird community structure changed in the region during these two periods.

FI could also be used to identify the presence or spatial extent of transition zones when moving from one spatial region to another, though we lacked data of sufficient spatial resolution to test this in our BBS case study. There is no limit in the size of the area (global, national, regional, city, or community), nor in the number of sampling sites used to capture the area. While a higher resolution is ideal, even sparse datasets afford the ability to capture behavior useful for assessing aggregate spatial (or temporal) patterns and trends. The case studies presented demonstrate the utility and versatility of the method through its ability to detect patterns in both continuous and discrete data. Note that while the data resolution in the initial simulated cases was much higher than the data used and generated for the breeding bird data, the Fisher information trends were distinctive and comparable. For example, Figure 2a,b and Figure 5b,c show that the method successfully identified patterns and trends (e.g., homogeneous and heterogeneous) in both relatively high and low(er) resolution cases. The case studies were used for illustrative purposes, as they served merely to highlight the possible uses of spatial Fisher information in an ecological context, rather than draw any ecological conclusions.

Furthermore, an application to multivariate data highlights the core strength of the method in capturing distinct trends in the index based on patterns in the underlying data. This is particularly important for the complex problems we face today, where drivers and management options are unknown or difficult to identify (e.g., harmful algal blooms). Future work includes exploring other distance approaches (e.g., nearest neighbor) or adding a spatial autocorrelation weighting factor to test the proximity between points. It would also be useful to examine the impact of the reference location (e.g., min vs. max latitude and longitude, closest to a particular feature) and to evaluate other approaches for estimating measurement uncertainty. Measurement uncertainty is a universal issue for data collection efforts, with data accuracy information often not being provided. Accordingly, it is critical that approaches be developed to handle this uncertainty. As noted in Section 2.1, when developing the computational approach for Fisher information [58,70], strategies were developed for estimating uncertainty by using the variation (e.g., standard deviation) of the measured variables in a similar system or in a relatively stable portion of the variables from the study dataset, as an approximation of measurement uncertainty. In this study, we used the range of simulated homogeneous data as a proxy for stable dynamics; however, as in temporal studies, the use of a “stable” (relatively low standard deviation) region in the raw dataset may preclude the need for a proxy.

Other forthcoming activities involve applying the method to other datasets (e.g., human, natural, social), particularly where there are known spatial shifts, comparing the index results to other approaches (e.g., principal components analysis, Moran’s I, early warning indicators), finding a means of combining both space and time into the assessment, and developing methods to identify which variables drive changes in the index to facilitate identification of management options.

This paper is a proof of concept and serves as a springboard for extending Fisher information to geospatial assessments. There are many questions left to be answered, yet this effort demonstrates a method that could provide a valuable tool for mining spatial data to detect latent patterns and signals in complex systems.

## Figures and Tables

**Table 1:** Patterns, parameters and expected FI for the simulated case studies.

Pattern	Variable Dynamics	Simulation Parameters	Expected FI
Homogeneous (HoG)	Relatively stable	HoG: mean (μ) = 50, STD (σ) = 2	FI→∞ (8)
Heterogeneous (HeT)	Highly variable	HeT: μ = 50, σ = 20	FI→0
Half and Half (HnH)	Half stable and half variable	HnH: Half HoG and Half HeT	FI→0 & FI→∞
Patch	Distinctly different patterns in a particular section	HoG with a HeT region	FI→∞ around edges and FI low toward the center

**Table 2:** Route details and breeding bird survey data sorted by Haversine distance (H.dist). The raw and simulated total species (TS) and total population (TP) data for 1990 are shown.

				Raw		HoG		HeT	
Route	Lon	Lat	H.dist	TS	TP	TS	TP	TS	TP
15	−93.34	30.01	49.49	44	691	49.92	834.73	35.87	1523.52
106	−93.02	29.77	59.60	43	1290	52.27	883.70	63.92	153.10
16	−93.32	30.83	97.00	61	613	52.51	731.16	31.75	825.01
34	−92.45	30.08	98.51	37	1489	51.68	1041.32	54.66	791.37
31	−92.23	29.85	106.56	56	1281	46.93	794.17	51.85	775.60
122	−93.50	31.07	108.75	41	506	49.87	790.54	74.38	468.32
113	−92.43	30.65	119.70	35	1557	50.57	823.04	50.43	640.17
14	−92.46	30.76	123.33	72	1063	58.23	879.98	32.06	435.44
30	−91.88	29.72	126.53	47	715	55.19	821.39	55.90	1044.21
11	−91.82	30.06	133.99	44	1157	52.26	876.80	43.09	828.21
37	−93.57	31.67	148.55	56	651	46.67	877.61	60.41	833.15
905	−91.64	30.37	151.11	59	1620	61.81	826.36	49.43	781.67
119	−92.96	31.56	151.78	60	743	57.15	808.26	50.15	1747.23
33	−91.51	30.40	158.82	62	1489	52.50	904.18	63.54	769.14
20	−92.30	31.46	165.96	47	494	57.40	1002.60	76.91	319.07
105	−91.21	29.70	166.57	52	993	52.03	1158.78	24.09	1127.89
12	−91.51	30.87	173.17	67	1240	55.97	852.82	57.46	664.27
27	−93.97	32.07	174.56	65	794	53.39	909.53	88.35	391.30
903	−91.20	30.41	176.76	54	1299	49.47	902.70	65.62	1671.22
17	−91.67	31.19	178.09	55	941	53.46	705.92	56.06	550.18
3	−90.92	29.90	184.86	53	544	56.39	847.28	91.87	683.87
29	−90.59	29.55	203.67	47	728	48.36	936.93	26.17	280.84
128	−93.48	32.55	209.80	54	644	54.00	1018.93	64.31	1059.71
125	−92.35	32.31	213.66	59	581	51.46	772.81	43.86	1380.79
32	−90.73	30.86	213.72	60	642	47.21	958.19	41.02	810.06
26	−92.62	32.46	216.65	47	538	50.43	934.38	37.17	997.79
9	−90.51	30.70	221.84	56	579	55.57	1008.05	56.33	374.07
18	−91.44	31.98	225.42	49	929	51.29	941.20	50.20	973.77
4	−90.10	29.69	233.14	55	1322	50.62	1071.73	46.51	1102.13
10	−90.25	30.88	240.76	65	621	57.23	892.86	35.60	1707.91
208	−89.85	30.27	252.32	52	493	54.61	780.47	65.70	432.20
38	−91.43	32.49	252.94	41	1007	54.56	876.27	66.42	1163.80
39	−92.28	32.95	255.62	57	650	55.31	908.47	41.62	1036.88
